# Intracranial Dissemination of a Cervical Sarcoma in a Young Patient With a Ventriculoperitoneal Shunt: A Case Report

**DOI:** 10.7759/cureus.93631

**Published:** 2025-10-01

**Authors:** Diana Lucas, André Gomes, Josué Pereira

**Affiliations:** 1 Neurosurgery, Centro Hospitalar Universitário de São João, Porto, PRT; 2 Pediatric Neurosurgery, Centro Hospitalar Universitário de São João, Porto, PRT; 3 Neurosurgery, Faculty of Medicine, University of Porto, Porto, PRT

**Keywords:** cervical sarcoma, intracranial ascending metastization, pediatric hydrocephalus, subcutaneus tissue path, ventriculoperitoneal shunt

## Abstract

Ventriculoperitoneal shunt (VPS) related metastization of intracranial tumors to the peritoneal cavity is an uncommon but a well-recognized complication. Ascending spread of tumors, located in the peritoneum or near the subcutaneous path of the VPS, is an exceptional rare event.

We present a very rare and complex case of intracranial dissemination of cervical tumor in a three-year-old young patient with a past medical history of hydrocephalus-related post-endovascular treatment of Galen vein aneurysm through a ventriculoperitoneal shunt catheter implanted when the patient was five months old.

## Introduction

Ventriculoperitoneal shunt (VPS) refers to a device divided into an intraventricular catheter, often referred to as the proximal catheter, a valve mechanism, and a distal catheter implanted in the abdominal cavity. The VPS implantation represents the most common procedure for cerebrospinal fluid shunting and is used to treat hydrocephalus. However, despite being highly effective, they are prone to complications [1].

An uncommon but well-recognized complication refers to the spread of intracranial tumors to the peritoneal cavity. It has been described in patients with medulloblastoma, germinomas and glioblastoma [1,2]. However, ascending spread of tumors located in the peritoneum or near the subcutaneous path of the VPS is an exceptionally rare event, with only a few cases described in the literature [1,3,4].

We present a case of intracranial dissemination of cervical sarcoma in a young patient with a VPS previously implanted to treat hydrocephalus-related to a Galen vein aneurysm.

## Case presentation

The patient was a three-year-old child with a long past medical history. At five months of age, he was diagnosed with a Galen vein aneurysm, posteriorly submitted to endovascular treatment. Approximately one week after embolization, he developed secondary obstructive hydrocephalus. Endoscopic third ventriculostomy was not performed because of anticipated technical difficulties and associated risks, namely intra-operative aneurysmal rupture, and implantation of fixed-pressure VPS was decided.

**Figure 1:**
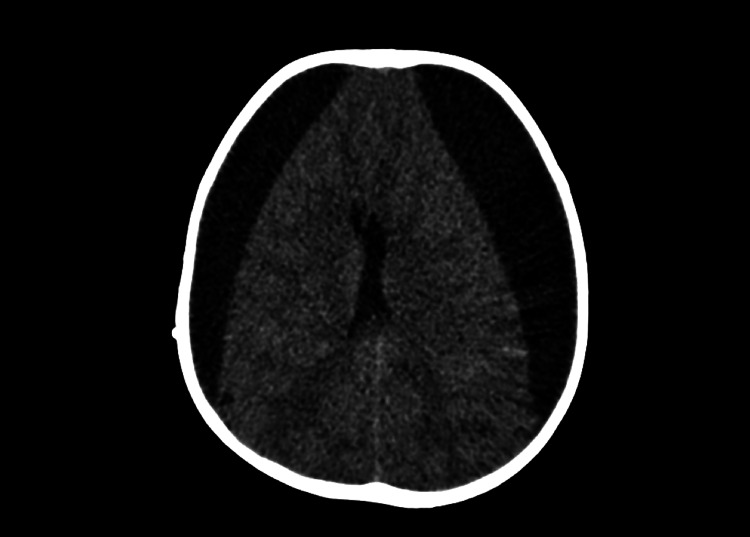
Image of bilateral subdural hygromas on head CT scan

He went well until June 2022 when he was brought to our institution with complaints of headache, anorexia and irritability. On head computed tomography (CT) at admission, he had large bilateral subdural hygromas (Figure 1), which already were documented in a previous follow-up head CT, but showed increased volume, with subsequent mass effect causing herniation of left temporal lobe. Due to clinical deterioration and image progression, urgent bilateral burr-hole drainage was performed, resulting in post-operative improvement. The patient was subsequently discharged.

A few days later, the patient returned to our hospital with the previous complaints, with imaging showing recurrent hygromas. The patient underwent burr-hole drainage and subsequent replacement of the VPS with an adjustable pressure shunt. His clinical condition improved, and he was discharged on the ninth postoperative day.

**Figure 2:**
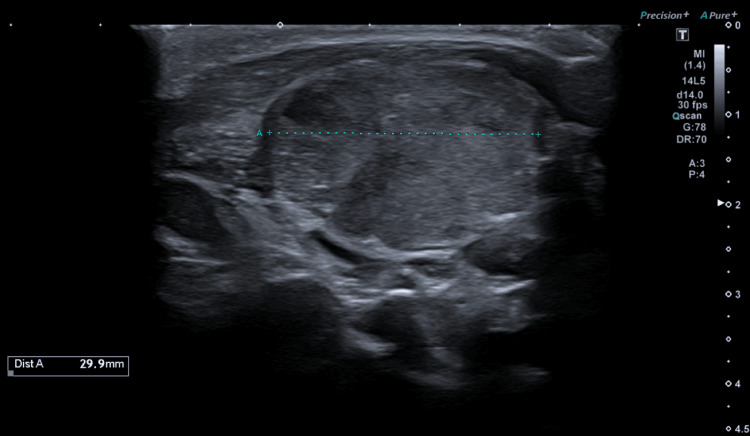
Ultrasound image of the right cervical swelling showing a large cervical mass (45x29.9x41.7 mm).

However, the patient was readmitted with headache and irritability, and pressure adjustment of the valve pressure was performed. During the stay in the hospital, he developed a neck and cranial swelling, along the shunt path, and a fever. Ultrasound of cervical swelling identified a large cervical mass, consistent with an adenopathy conglomerate (Figure 2). The physical and ultrasound findings were interpreted as an infection process, associated with VPS, and the patient was submitted to new shunt revision. At the revision surgery, we found gray, gelatinous material around the valve, distal catheter and burr-holes entrance, and it was presumed to represent a foreign body reaction. No purulent material was found and microbiological examination of the samples collected at surgery was negative. VPS was removed and the patient was placed on external ventricular derivation.

**Figure 3:**
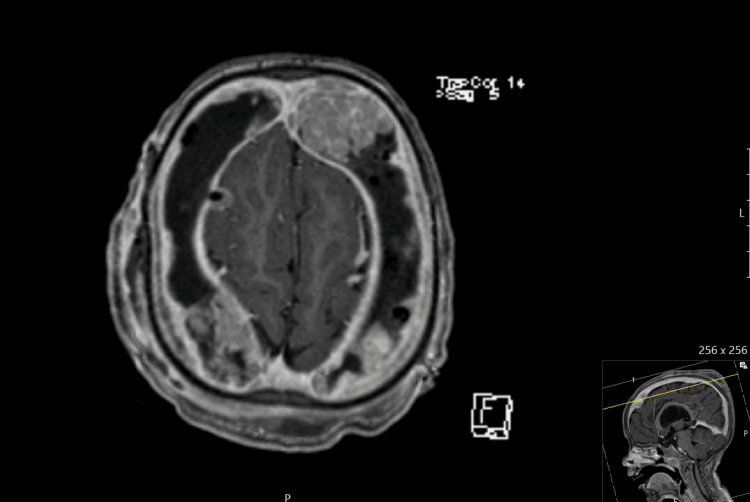
Contrast-enhanced axial T1 MRI image showing subdural space occupied with multiple nodular masses.

The patient was later evaluated with magnetic resonance imaging (MRI) to assess the possibility of intracranial infections. Instead, bilateral subdural nodular masses were found, which raised the hypothesis of a lymphoproliferative process (Figure 3). These findings weren't present on head CT that the patient performed six days before revision surgery.

**Figure 4:**
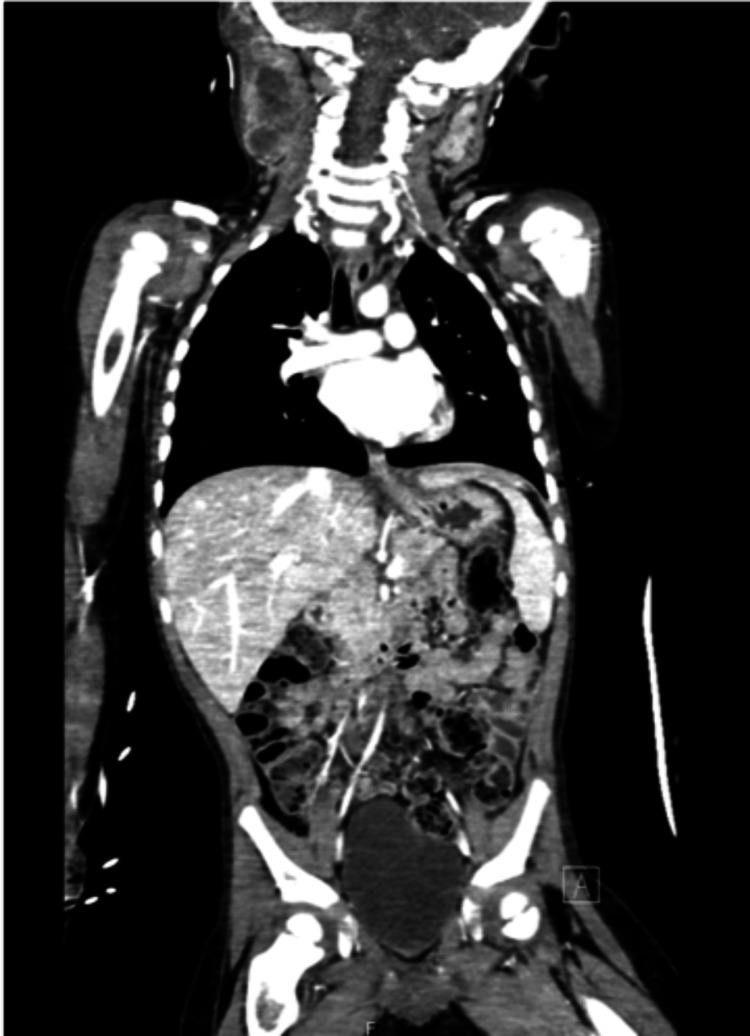
Head, cervical, thoracic, abdominal and pelvic CT images showing a single large cervical mass.

Further workup with neuro-axis MRI found a nodular mass in the most cranial aspect of the dural sac. Thoracic, abdominal and pelvic CT demonstrated a large cervical mass (Figure 4), which was further characterized by MRI. No other lesions suggestive of metastasis were found. The patient underwent an ultrasound-guided biopsy of the cervical mass. Histopathologic assessment revealed a sarcoma composed of round cells with myxoid/mucoid background, and CD99 positivity, suggesting Ewing origin in plasma-cell like variant sarcoma.

Because of enlargement of cranial end cervical masses, and worsening of clinical status, the patient started chemotherapy with temozolomide, irinotecan and vincristine. 

## Discussion

Although not a common complication, there is evidence that cerebrospinal fluid (CSF) shunts can provide a route for systemic spread of nervous system tumors. Peritoneal spreading is, by far, the most common site of metastization because of drainage of CSF containing tumor cells to the abdominal cavity [5]. Spreading of cranial tumors to the scrotum [6] and pleura [7] has also been reported.

Nonetheless, the possibility of retrograde metastatic spread of tumors has been reported [1], occurring more frequently along the subcutaneous path of the VPS, and rarely it can spread intracranially [8-10]. We present a very rare case of ascending dissemination of a cervical sarcoma into subcutaneous path and intracranial subdural space along the proximal part of the peritoneal catheter. To our knowledge, there are only two published cases of intracranial metastasis associated with VPS. Heuer et al. present a similar case of a young child with a cervical and an intracranial sarcoma, and presumed an ascending spreading of the tumor along the subcutaneous path, although the first mass to be detected was the intracranial one [7]. Eralp et al. describe a case of leptomeningeal dissemination of ovarian cancer by the CSF across the lumen catheter in the opposite flow of fluid, not across the subcutaneous path of the catheter [3].

Three possible routes of retrograde spread have been described by Frantzias et al.: (a) intraoperative dissemination, (b) dissemination through the subcutaneous path of the shunt and (c) intraluminal dissemination via the CSF [4]. In our case, given the location of the primary tumor, we think that dissemination along the subcutaneous path of the distal catheter of the VPS occurred. However, even if remote, the possibility of intraoperative dissemination during the subcutaneous passage of the distal catheter should be considered, even though no evidence of disease was present at the time of shunt replacement.

Furthermore, it is important to highlight that the occurrence of hygromas and subsequent shunt dysfunction could be an early sign of intracranial spread of tumor cells, even though no evidence of systemic disease was apparent at the time of initial presentation nor was it evident the presence of signs suggestive of intracranial tumor spread upon initial neuroimaging studies.

## Conclusions

This clinical case highlights the risk of ascending dissemination of neoplastic disease in patients with VPS, along the subcutaneous path of the catheter, into the intracranial space. It is important, in such cases, to consider that shunt dysfunction can occur due to such dissemination and careful imagiological workup should be employed.
